# Sport for Development and Global Public Health Issues: A Case Study of National Sports Associations

**DOI:** 10.3934/publichealth.2017.3.240

**Published:** 2017-05-26

**Authors:** Davies Banda

**Affiliations:** University of Edinburgh, Moray House School of Education, Institute of Sport, PE and Health Science, St Leonard's Place, Edinburgh, EH8 8AQ, United Kingdom

**Keywords:** HIV/AIDS, multisectoral approach, sport-for-development, governance, HIV/AIDS mainstreaming, Top-down, Bottom-up, nongovernmental organisation, policy network theory

## Abstract

Sport is widely recognised for the contribution it can make to international development goals. More specifically, the value of sport as a tool for development gained its impetus through the HIV/AIDS pandemic in sub-Saharan Africa. The institutionalized relationship between sport and development has mainly focussed on sport-for-development (SfD) non-governmental organisations (NGOs). This study proposed to examine the response of National Sports Associations (NSAs) towards the multisectoral approach for HIV/AIDS prevention in Zambia. The study draws on lessons learnt from how NSAs within a resource-scarce or low-income country responded to a health pandemic. While public health was previously a state and health sector preserve, the impact of HIV/AIDS pandemic influenced not only the way that a pandemic is managed but also other public health issues. A case study approach was adopted comprising of three National Sports Associations (NSAs) as units of analysis. The study utilised semi-structured interviews, documentary analysis and field observations to gain perspectives on how each NSA mainstreamed and implemented work-based health programmes. Using governance and policy network theories, the paper discusses each NSAs' role in the governance and implementation of a multisectoral approach to a health pandemic. The findings identified lack of engagement of sports agencies at strategic decision-making level, marginalisation of sport by other sectors, and variations in implementation patterns among sports agencies. Further findings indicate that lack of resources among government sport agencies or departments limited their involvement with other state or non-state actors in strategic level meetings or health policy networks. Resource-scarce conditions placed limitations on the political steer of state actors while non-state actors with foreign resources attracted collaboration from other public health policy networks.

## 1. Introduction

Sport is widely recognized for the contribution it can make towards social change. Although social change can be either positive or negative [1], whenever sport is used to achieve development outcomes, the emphasis is on positive social change. Hence, internationally, trends formalising the status of sport as an agent of social change for international development strategies or initiatives have continued to increase[2],[3]. Darnell and Black [4] reinforce that, sport as a social, cultural and political phenomenon and institution, has significant implications for development and peace. Despite sport in general having a lengthy history of servicing “social development” objectives [5], the notion of sport-for-development (SfD) is depicted by Kidd [6] as a “new social movement”. The growth in significance of this new movement can be attributed to three key developments by the United Nations (UN): the appointment of UN's first Special Advisor on Sport for Development and Peace in 2001; the establishment of the UN's Inter-Agency Task Force on Sport for Development and Peace in 2002; and establishment of UN's Office for Sport for Development and Peace (UNOSDP) in 2005 [5]. Furthermore, growth has resulted from an increase in the prevalence of foreign funding for SfD programmes in low-income countries. The funding sources consisted of international development agencies, international sports federations, charities and private sector organizations [6] located in high-income countries.

Other significant contributions towards the growth of the SfD sector include: the inclusion of SfD in strategic plans to achieve development outcomes by both the United Nations [7] and the Commonwealth Secretariat; and the exponential growth in number of SfD non-governmental organisations (NGOs) [9]. SfD NGOs are defined as those organisations that use sport to help individuals and/or communities achieve non-sporting, personal and/or social development outcomes [5]. SfD NGOs have been instrumental in linking sport and global health campaigns such as the campaign to tackle the spread of the *human immunodeficiency virus* (HIV) which causes the *acquired immune deficiency syndrome* (AIDS). Coalter [10] points out that HIV/AIDS provided the impetus for the growth of the SfD movement. In practice, sport settings have served as conducive environments through which information about the virus can be disseminated to influence positive personal and social behaviours that reduce incidences that lead to infection [11]. Hence, in Zambia, the social setting of this study, sport forms part of the national strategies to combat HIV/AIDS under the national multisectoral response [12].

Despite its recognition as an agent of social change, scholars [3],[4],[12] within the SfD sector are cautious of how sport at times is considered as a panacea. This is particularly evident in how sport interventions articulate their missions, objectives or expected outcomes [4],[13]–[15]. Other scholars researching sport for social change schemes alert practitioners and policy makers of the fact that it is not sport per se that is responsible for particular outcomes but the ways in which sport is implemented. For example, Spaaij [13] warns that the transformative capacity of such schemes “can only be realised within a personal and social development approach and not by merely offering sport activities”. Whilst several academic studies [16]–[18] have researched the link between sport and HIV/AIDS development outcomes, this study focussed on examining the response of National Sports Associations (NSAs) towards the multisectoral approach for HIV/AIDS prevention in Zambia. The multisectoral approach is considered an effective approach for building a concerted effort to help achieve HIV/AIDS policy goals. Such a response aligns with the Ottawa Charter for Health Promotion 1986 which views health promotion as not just the responsibility of the health sector but reaching out and engaging directly with sectors traditionally seen as remotely positioned to deal with health matters. Dixey [19], however recommends the Alma Ata Declaration of 1978 aimed at developing countries over the Ottawa Charter which targeted industrialized states.

As the SfD movement is considered to be in its infancy stage, critical theorists [3],[4],[20] within and beyond the SfD movement have questioned the lack of an evidence base. Indeed, there is currently little definitive evidence to demonstrate the role of sport as an effective tool in achieving international development outcomes. Coalter [19] encourages SfD programme designers and evaluators to strongly consider the underlying theories and mechanisms or processes in order to overcome commonly held assumptions that sport is inherently beneficial. It is vital to understand the necessary conditions or settings that enable sport programmes to bring about the desirable change [19],[21].

The focus of this study is on the role played by National Sports Associations (NSAs) within public health policy strategic forums. In comparison to SfD NGOs, the ethos of NSAs is sports for sports' sake [22]. NSAs have a mandate to deliver sporting outcomes related to grassroots participation and elite performance. In Zambia, NSAs have neglected grassroots sport due to insufficient funding from central government. Instead, as will be elaborated later, SfD NGOs that emerged as a result of neoliberal reforms deliver both non-sporting and sporting outcomes. Notwithstanding their proportionate dependency on foreign donors and development outcomes ethos, SfD NGOs significantly contribute towards grassroots sport [23],[24]. They tend to cover government and NSA community sport inefficiencies and have positioned themselves within civil society sector. While there is a growing body of literature on partnerships between SfD NGOs and health agencies, this is the first research specifically assessing the role of NSAs within the HIV/AIDS multisectoral approach.

## 2. Background to HIV/AIDS Governance and Sport in Zambia

When the first case of HIV was diagnosed in 1984, the response lacked high-level political commitment and strategic management [12]. The governance of HIV/AIDS was initially restrictive to health sector agencies only. Non-health sector actors, in particular non-state actors such as civil society organisations (CSOs) were marginalised from health-related service provision. State actors dominated health agenda setting, policy formulation and implementation. However today, a broad range of non-state actors from the civil society are involved in delivering health services. HIV/AIDS governance research [25] demonstrates how approaches to decentralise governance and devolve power to civil society has helped build a concerted effort. State dominance in public policy making has eroded in many sectors.

NGOs gained recognition as partners in health care provision in 1993 when changes demanded by the World Bank in health provision called for the inclusion of non-state actors to promote diversity and competition [26]. NGOs or CSOs gained favour over state actors from the donor community resulting in side-lining of state actors [27]. Reasons cited in favour of NGOs within development literature include attributes of NGOs as: encompassing or representative of local/community leadership; effective response to local needs due to their close proximity; and that such proximity enables aid to reach the intended target group [28]–[30]. The preference for NGOs and availability of foreign donor resources resulted in the proliferation of NGOs within the HIV/AIDS policy environment in Zambia [31]. Other quasi-nongovernmental sports agencies such as NSAs were ineligible to access foreign donor funds. The ineligibility of NSAs marginalised their involvement in international development strategies whilst that of SfD NGOs was strengthened. The marginalisation of quasi-nongovernmental organisations is linked to neoliberal reforms that widely considered African states as corrupt and inefficient [27]. Hence, much of the funding was channelled through NGOs, particularly international NGOs.

While the state played a minimal coordinating role, the geographical limitation of NGOs [27] as delivery partners of global health initiatives hampered efforts to address the HIV/AIDS pandemic [25]. In addition to their geographical limitation, the competition for foreign resources fuelled antagonism among NGOs, and subsequently fragmented approaches aimed at similar target communities [11],[32],[33]. In response to the uncoordinated and fragmented approaches between state and non-state actors, the establishment of the National HIV/AIDS/STI/TB Council (NAC) in 2002, helped bring about coordination. The establishment of the NAC formed part of the crucial prerequisite stipulated by UNAIDS in the “Three Ones” principles [34] for governments namely: *one AIDS action framework; one national AIDS coordinating authority; and one country-level monitoring and evaluation system.* In support of the establishments of the NAC, Morah and Ihalainen state three key reasons: (1) the need for a robust multisectoral response due to the complexity of combating the HIV/AIDS pandemic; (2) the need for a multisectoral response consisting of state and non-state actors opposed to a dominant traditional public-health paradigm which was out of tune with the challenges of combating HIV/AIDS and; (3) the lack of authority of Health Ministries to command other government departments [35].

Re-orienting of the response to HIV/AIDS governance meant that quasi-nongovernmental agencies like NSAs, once marginalised, were now eligible to receive donor funds under the multisectoral approach. However, since foreign donors still wield huge economic resources, the power to set agendas, priorities and preferred delivery mechanisms largely lies with them. As earlier discussed, the absence of a compelling evidence base in support of the SfD movement causes concern for potential funders to adopt sport as a development tool. Zambia's unique political, economic and social country background [24] has made it a prime arena for researching the role of SfD purposes. The trajectories provided by the local SfD NGOs' involvement in global health initiatives offers us insightful lessons linking sport with international development outcomes.

Most of SfD research in Zambia has focussed on micro-level analysis of SfD NGOs or cases of sport and HIV/AIDS schemes at community level. This is a meso-level analysis of NSAs [36],[37] focussing on the involvement of national federations in public health policy. The rationale for conducting this study was to examine the role played by three meso-level National Sports Associations (NSAs) in the governance, policy making and implementation of public health policies at national level. The research objectives were twofold: first, to investigate the involvement of NSAs in health-pandemic governance under the HIV/AIDS multisectoral approach; and secondly, to analyse in-house HIV/AIDS mainstreaming the policy making practices within each NSA. Governance theory and policy network theory are discussed next in relation to the influence of global health governance [38] and global health initiatives (GHIs) [39] on the governance of HIV/AIDS at the national and subnational level.

### Theoretical Insights

#### a) Concepts of Governance in Development

The extensive use of the word “governance” in development literature over the past decade denotes a shift in focus from government to governance. Governance denotes new modes of governing that are not in the form of hierarchical control but much more of cooperative processes of governing between state and non-state actors [40]. Rose comments that today, political leaders attempt not to govern by way of centralised bureaucracies, but through self-organising networks resulting from interactions and interdependencies of both state and non-state actors [41]. Rhodes termed this as “hollowing out of the state” [42] or the decentring of government [43]. Rhodes also refers to it as “governance without government” [44]. Public policy making and grassroots delivery of public health services or programmes in Zambia was mainly a preserve of health sector civil servants. Today, public health service delivery and administration at grassroots level has undergone transformations to new modes of governance in which the state has ceased to be the “cockpit from which society is governed” [45].

Despite new forms of governance centring on the reduced role of the state, Bell and Hindmoor argued that governments still play a central role in making new governance a possibility or option for governing [46]. In terms of the multisectoral approach towards a health-pandemic, the role of government is still crucial. This is so as government can set the policy agenda, positioning the policy matter at the core of policy making and implementation, and involve other actors in a concerted effort [28],[47]. Such shifts from government to new forms of governance have been conceptualised by Howlett, Rayner, & Tollefson into Hierarchical and Plurilateral governance [48]. Table 1 depicts hierarchical governance as associated with top-down systematic planning and implementation of policies, a system in which political power favours state actors. The state's machinery to enact policy is legitimate and authoritative but lacking flexibility and innovation [25] compared to what may be evident within society-centred, plurilateral governance. In plurilateral governance, a wide range of non-state actors exercise power, engage in decision making, and are governed by a jointly agreed code of practice.

**Table 1.** Government to Governance Multi-dimensional Conceptualisation.

	Hierarchical Governance	Plurilateral Governance
**Dimensions**	State-Centric	Society-Centred
**Political Practices**	State actors exercise power	Non-state actors exercise power
	State directed policy networks	Issue networks
**Institutional Structures**	Formal institutions	Informal institutions
	Formal consultation	Wide range of non-state actors
	Clear lines of authority	Shared decision making
**Regulatory Techniques**	State centred	Market oriented
	Tight regulatory control	Society centred
	Legitimate and authoritative decisions	Code of practice
		Voluntary agreements

Adapted from Howlett, Rayner, Tollefson (2009)

#### b) Policy Network Theory

The HIV/AIDS pandemic in Zambia has significantly transformed the public health policy landscape in what was before a state actor dominated space. Today, the public health policy space is characterized as an interdisciplinary and collaborative space opposed to the dominated space found in state bureaucracies or hierarchies. Neoliberal approaches have influenced such policy spaces, enabling state and non-state actors to interact and influence policy. The days of a monolithic state controlling the policy making process are long gone. Hence, policy network theory provides analytical purchase to examine the public health landscape as “involving a diversity of actors who are mutually interdependent” [49]. It is for this reason that the use of policy network theory, its typologies and impact on the policy process are utilised to analyse national public health policy changes. The utilisation of network theory has been conducted mostly in high income countries where a proliferation of non-state and state actors frequently interact. Its application for public health policy analysis within a low-income country still holds strong due to the pluralism that prevails in Zambia. The HIV/AIDS policy environment has a multiplicity of state and non-state actors operating interdependently.

Using policy network theory to examine the governance structure of HIV/AIDS under the multisectoral approach, the theory enlightens us about the following: why some actors have limited participation in decision-making; why some actors enjoy more privileges than others; why some issues get included or excluded from the agenda; and why the understanding of resource exchange by some members maximise their influence to attain their goals [50]. Therefore, the policy network approach has analytical purchase.

The use of the term “policy network” has featured a variety of models which sometimes causes confusion because the same term had previously been used to mean or refer to different interactions or interests. Marsh and Rhodes' [50] classification adopted by this study divides policy networks into two distinct categories: policy communities and issue networks. These two categories are differentiated based on membership, integration, resources and power. Marsh and Rhodes describe a policy community as having highly-limited access to its membership, tightly integrated, high frequency of interaction, share basic values, well-organised and where all members have resources to exchange. By contrast, an issue network is characterised as loosely organised, infrequent interaction of the large number of members involved, each of whom hold diverse values and little continuity.

## 3. Methodology

A case study approach [51] utilising qualitative methods of data collection was adopted for this meso-level policy analysis. As meso level organisations, these organisations operated at the national policy level sitting between the macro level (state agencies–Department of Sport) and micro level (community based organisations such as the SfD NGOs). The study utilised multiple cases purposively selected to examine the role played by sports organisations within multisectoral public health policy networks. The case study selection criteria was mainly based on the characteristics of the sporting code focusing on: nationwide participation rates or geographical coverage; high significance within Physical Education curriculum and school sport programmes; gender representation; and level of organised competitive events on school and community sport calendars across local, regional and national levels.

The selected National Sports Associations (NSAs) were: Football Association of Zambia (FAZ); Netball Association of Zambia (NAZ); and the Zambia Basketball Association (ZBA) (football–mostly male; netball–mostly female and basketball–both male and female representation). Interviews were conducted by the principal researcher between 2010 and 2014. Interviewees were provided with information sheets via email. Verbal and written consents were obtained before proceeding with data collection. Data presented here are drawn from eleven (11) semi-structured interviews conducted with: NSA's President or the General Secretary; NSA sport development officers; government officials representing the Department of Sport Development (DSD) in the Ministry of Sports, Youth and Child Development (MSYCD); National Sports Council of Zambia (NSCZ) officials; and lastly, senior officials from the National AIDS Council (NAC). Sports Association and Sports Department senior official interviewees were purposively sampled based on their seniority and their involvement in the day to day handling of policy issues within each sport association and government department, respectively. An interview guide was used to make the process of interviewing a number of different people more systematic and comprehensive [52].

Other data collection methods included documentary analysis which focussed on public health policies, national HIV/AIDS policy documents such as the National HIV/AIDS Strategic Frameworks (NASF) 2005–2010 [53], NASF 2011–2015 [54] and in-house government agency and NSA strategic development plans. These were used to develop a thorough understanding of the response to the adoption of the HIV/AIDS multisectoral approach. A prior “detailed examination of documents” [55] mentioned above facilitated the formulation of items for the semi-structured interview guide. Overt observations at policy network meetings and sports competitions were conducted and these also contributed towards snowball identification of actors within policy networks. Method triangulation of data [56] was achieved through the various sources of data collection regarding the same phenomenon mentioned above. Interview transcripts and field notes were analysed through an iterative and inductive process [60]. This consisted of initial coding and then going back and forth through the coded data, connecting and interrelating data to enable interpretations to be formed [57]. Both theories of governance and policy network were utilised in the process of coding, looking for patterns and the identification of themes until saturation. Ethical approval for this study was obtained from the Faculty ethics committee at the researcher's university and further reviewed by the National AIDS Council in Zambia.

## 4. Results and Discussion

### 4.1. Involvement in National and Subnational Coordination Structures

Global Health literature [58] highlights how governments experiencing high HIV/AIDS prevalence were advised to adopt multisectoral approaches. By the time Zambia was establishing the National AIDS Council (NAC), the International Monetary Fund (IMF) and World Bank's structural adjustment programme (SAP) had already weakened state investment in health services [5],[24]. With a weak public health sector, the involvement of non-state actors was inevitable. The NAC established subnational level structures consisting of state and non-state actors. Government sport departments and non-state sport actors at national, provincial and district level were eligible for membership of these subnational structures [12]. Their eligibility was implied by a Senior NAC Official who elaborated on the tenets of the multisectoral approach:

I think really what worked for us is to have a coordination mechanism at district, provincial and at national level. That way it's easier for us to monitor who is doing what at a micro level and at a macro level…the multisectoral approach for the Zambian context means that different sectors bring to the HIV/AIDS response their skills, their resources, and their comparative advantage.

The findings revealed a wide range of stakeholders comprising of government agencies, civil society organisations, and the private sector. This reflected the new governance approaches of health pandemics, proving HIV/AIDS could not be tackled by the health sector alone [41]. These actors were representative of “society-centred governance” mechanism [51].

The composition of the NAC was indicative of the multisectoral response at national level as it comprised of four Permanent Secretaries positioned in government ministries responsible for: Education; Health; Community Development and Social Welfare; and Sport, Youth and Child Development. The rationale for the inclusion of each of the four policy executives were as follows:

- Ministry of Education–education as a “social vaccine” within school settings
- Ministry of Health–the NAC is under the authority of the MoH as a semi-autonomous organisation
- Ministry of Sport, Youth and Child Development (MSYCD)–HIV/AIDS impacts the household more severely than any other unit. Children and young people suffer the impacts of HIV/AIDS considerably more than other groups [12].

Of interest to this study among these four ministerial state actors was the MSYCD which consists of three departments, namely: Department of Sport Development (DSD); Department of Youth Development (DYD) and Department of Child Development (DCD). Notwithstanding that all three departments fall within the remit of the MSYCD Permanent Secretary (PS), only youth and child policy concerns were highlighted by a NAC official:

It was deliberate that as National AIDS Council, the youths have a permanent seat on the council so that we work together with them to see how then do we address the challenges that the youths are facing and how HIV/AIDS is also affecting the progress within the youths.

Further marginalisation of the value of sport in interactions between the MYSCD and NAC were evident when NAC representatives attended strategic meetings at the MSYCD office headquarters. Rather than engage all three departmental directors, a DSD Senior official reiterated that the NAC's significant focus was on the roles of the DYD and DCD in matters related to youth and child welfare:

…actually, the NAC Director of Multisectoral Approach came to see the Child Department and then as we were talking, I think someone said, “no by the way we have got Sports Department [also within the MYSCD]”.

The marginalisation of the Director for the DSD by the NAC was indicative of the lack of integration of sport within HIV/AIDS implementation plans. An exclusion like that above from formal strategic meetings continues to exacerbate the lack of integration of sport into development activities. The sport sector needs to integrate with planning for development outcomes at the national level and also with mainstream development agencies in order to make significant contributions towards evidence production. Policy network theory [46] provides some analytical purchase in relation to the grouping of the DYD, DCD and NAC Director of Multisectoral Approach as forming a problem specific entity. HIV/AIDS prevention and care for young people was a specific policy area within which the NAC intended to design collective action between NAC officials and the two departments. Notwithstanding the popularity of sport among young people or its recognition as an agent of social change, the NAC overlooked sport's value to achieving development outcomes.

Consequently, within meso-level policy networks, the study revealed less engagement of government sport agencies (DSD), semi-governmental (National Sports Council of Zambia) and quasi-nongovernmental bodies (NSAs) in wider development activities. However, the emergence of SfD NGOs as part of the new brigade of youth services demonstrated integration of sport organisations into wider development agendas at micro level. SfD NGOs were active within NAC's subnational structures at provincial and district level, namely: Provincial AIDS Task Forces (PAFT) and District AIDS Tasks Forces (DATF) [5],[12]. Unlike the closed policy network between the NAC, DYD and DCD identified above, the PAFT and DAFT were open to both state and non-state actors to share practices within a public health policy space. Marsh and Rhodes' description of issue networks [50] fits these policy networks since they were loosely organised and had open membership as confirmed by a NAC official:

We all get invited and sometimes word gets round from NGO to NGO that there is going to be a district AIDS meeting every few months or so…all NGOs and government units get invited to talk and share about AIDS as long as they have a story

Marsh and Rhodes further stress resource dependency as a key feature of policy networks and that resources determine the level of hierarchy within networks. Despite the unrestricted access to the identified self-organising networks, lack of resources limited the involvement of most of the sports agencies from these information sharing, giving and policy shaping forums [16]. The study revealed that only SfD NGOs which had access to foreign resources engaged in these forums. All the three case studies (NSAs) were not involved in these open issues networks. Since NSAs are poorly funded by the government, they have been unable to frequently interact with key actors among the different HIV-focussed policy networks. Conversely, SfD NGOs despite their limited geographical spread nationwide interacted more frequently with non-sport actors within HIV-focussed policy networks. As meso-level organisations, the absence of NSAs from such networks, limited the influence sport as a sector could have upon the governance of public health policy.

### 4.2. Evidence of Mainstreaming Health Issues Among all the Three NSAs

Having noted the absence of NSAs within HIV-focused policy networks, the study sought to focus on internal organisational deliberations fulfilling the HIV/AIDS mainstreaming agenda [12],[57]. Further application of policy network theory was used to examine external interactions, cooperation and learning that NSAs utilised to facilitate the mainstreaming of HIV/AIDS, gender and human rights [56],[57]. This section is divided into two parts: a) Developing Institutional Policy Statement; and b) Networks, Conflicts and Policy Collaborative Communities

#### a) Developing Institutional Policy Statement

Institutional commitment to engage in policy making networks to inform the development of in-house HIV/AIDS policy varied among the three cases. In spite of the requirements within the national sport policy [59] to demonstrate in-house mainstreaming of HIV/AIDS, gender and human rights, the lack of resource commitment from the NSCZ and DSD hampered the consistency of their responses. Consequently, the lack of support exposed the gaps between policy rhetoric and practice as each NSA responded to HIV/AIDS mainstreaming differently causing inconsistencies within sport:

*We have a lot more capable doctors… they are not full time doctors of the national team, they work in hospitals so their work is very valuable now to the Football Association* (FAZ Interviewee–Personal Interview)

*It [HIV/AIDS] is there in our strategic plan, …What we said is that if we keep a child active through sports, we may one way or another try to move them away from these other vices that may bring their status or the health levels down. So in one way or the other HIV/AIDS is also incorporated …as a teachers or council officers, we have programmes at work* (NAZ Interviewee - Personal Interview)

*We [the ZBA] don't have a specific policy for [HIV/AIDS] but how we have gotten around the issue of not having a specific policy is to look at the [external HIV] programme itself. If it has to involve youths, we are assisted maybe through Ministry of Education guideline on behaviour change* (ZBA Interviewee - Personal Interview).

These variations were shaped by each NSA's internal capabilities, resources and external relationships with those in health policy networks. Football, the national sport, had more resources compared to the other two cases, ZBA and NAZ. Part of its human resource were medical personnel who belong to the FAZ Medical Committee (FAZ MedCom). This internal capability was utilised for policy guidance and implementation within FAZ. The FAZ MedCom was characterised by highly restricted membership and shared basic values [32] as medical professionals. Therefore, the FAZ MedCom brought a sense of organisational self-sufficiency in public health policy matters since members also belonged to other health-based policy networks.

Among all three cases, the Netball Association was the only NSA which had included the use of sport as a tool for *raising health awareness and supporting sustainable development* within its strategic development plans. Netball, being a female dominated sport, placed strong emphasis on mainstreaming of gender within its strategic development plans, a key aspect of addressing the HIV/AIDS pandemic. However, despite NAZ's clear intentions for mainstreaming HIV/AIDS and gender, no formal engagement in HIV/AIDS and gender policy networks were noted within this NSA.

The ZBA acknowledged its resource-poor conditions and utilised the various networks within the highly fragmented HIV/AIDS policy space to mainstream HIV/AIDS. Of interest was the acknowledgement by the association that its members possessed membership of multiple social entities. There was a sense of confidence that ZBA members had access to well-designed HIV/AIDS and gender mainstreaming programmes elsewhere such as educational institutions or places of work. Hence, developing institutional plans was seen as failing to grasp the policy rhetoric-reality gap faced by the NSA:

*We have not really stressed HIV and the reason is basically because…we don't have a specific budget that would look at those things* (Senior ZBA Official, Personal Interview).

Therefore, no formal policy on HIV/AIDS mainstreaming existed. In comparison to FAZ, both ZBA and NAZ were woefully under-funded by central government and did not have the same political power as FAZ to influence HIV/AIDS policy decision-making and implementation.

#### b) Networks, Conflicts and Policy Collaborative Communities

Within the sport and HIV/AIDS policy space in Zambia, non-state actors, the SfD NGOs, were confronted with animosity by government. The animosity developed from a perception that SfD NGOs were diverting foreign resources away from government [2],[18]. Before the advent of SfD NGOs in Zambia, foreign resources, mainly from Norway, funded a sport-for-all project in collaboration with the NSCZ. The implications of neoliberal approaches discussed above influenced the decision by a Norwegian development agency to start funding SfD NGOs and terminate a government sport-for-all project. This resulted in animosity between government officials and SfD NGOs. Subsequently, both the NSCZ and the DSD considered SfD NGOs as outsiders and not part of the legally recognised sport policy network of providers. It took almost two decades for the DSD to begin to recognise the significant role played by SfD NGOs at community level [5],[24]. SfD evaluation [10],[16] reports critiqued the fragmented approaches of delivering sport in Zambia, it brought a realisation to all concerned stakeholders that much can be achieved if SfD NGOs and the DSD/NSCZ worked collaboratively. SfD NGOs were instrumental in setting up collaborations that capacitated NSAs to deliver HIV/AIDS activities. Acting as development practice intermediaries SfD NGOs facilitated pathways for NSAs to utilise sport to address wider development agendas:

*We have worked with a lot of NGOs, with SCORE, with the Norwegians…we would bring people from the D.E.C–Drug Enforcement Commission, doctors, peer educators [who] would be able to speak to [national footballers] about HIV* (FAZ Interviewee–Personal interview).

*We have had involvement of CHAZ and Society for Family Health at our events* (Former ZBA President–Telephone Interview).

*We have partnerships with Sports in Action, NOWSPAR and EduSport Foundation. Through their [SfD NGOs] partnerships with other international organisations [UK sport and England Netball], they are able to relay health education through their workshops* (Personal Interview, NAZ Senior Official B).

SfD NGOs had links to a multiplicity of development sector actors enabling bilateral and multilateral collaborations involving NSAs, health sector HIV-focused NGOs and other varied civil society groups. Such collaborations were generally informal and closely matched the conceptualisation of plurilateral governance posited by Howlett, Rayner and Tollefson [51]. The wide range of non-state actors and informal relations also fitted the typology of issue networks posited by Marsh and Rhodes [50] and Heclo [60]. These collaborations were open interactions, with fluctuating access and consisting of members who had diverse views.

Of significant importance, regarding the instigation of these identified policy networks, were the restricted eligibility to foreign donor funding available only to SfD NGOs despite their geographical limitation. However, the attraction of NSAs to other actors was their countrywide geographical coverage. Hence, collaborative planning involving NSAs, the DSD and other non-state actors was essential since SfD NGOs were limited in geographical coverage during programme implementation.

A new policy community [50] in sport and public health was identified under the auspices of the Fédération Internationale de Football Association's (FIFA) sport and health initiative launched in Zambia in 2012. The “FIFA 11 for Health' global health programme focuses on providing school based health education in a football setting for children” [61]. FAZ spear-headed the policy community workshops which comprised of representatives from three ministries: Education; Health; and Sport, Youth and Child Development. This was the only formalised sport and health based programme under the leadership of a sport governing body. A senior FAZ official envisioned the FIFA “11 for Health” programme as a solution to the scarcity of resources encountered by all three ministries:

*The Ministry of Education, they are very capable …and we have spoken in that regard and that is why I feel once we bring the grassroots programme you know which is fully sponsored by FIFA it can bring change because, of course the Ministry of Education needs funding, we, FAZ, need funding, the Ministry of Youth and Sport needs funding* (Personal interview).

The FIFA “11 for Health” membership closely fitted Marsh and Rhodes' policy community as an “integrated community” [50] characterised by closed membership and shared interests in the health and wellbeing of young people. As a policy community, the programme's policy planning meetings were a strong fit of meso-level analysis of specific public health relations between state and non-state actors. Bring together three governmental ministries, the “11 for Health” programme in Zambia enabled mechanisms for coordination of public health policy and accountability:

*“we have put the programme to operate at both [levels], if there is no room during PE, they [teachers] can use the programme during sports but we would have loved to have infused it into PE, that is why we are using teachers who have PE on their timetables and able to use their PE lessons for this programme”* (Senior FAZ Official–Personal interview)

Revisiting the SfD limitation regarding the lack of an evidence base for sport as a tool for development, perhaps the composition of such policy communities as the “11 for Health” programme can enhance the gathering of evidence from different departmental/sectoral experts. Health programmes like the FIFA “11 for Health” demonstrate a shift from hierarchy (MoH) to heterarchy (multiplicity of actors in governance) [50]. While programmes such as these may redress the constraints posed by hierarchical organisation of government, their alignment to national health objectives is essential. SfD scholars have warned of the dominance of global North-orientated agendas [3]–[5],[62] which pose a risk of marginalising local needs and policy priorities. A failure to integrate the contributions of either local or foreign designed programmes into official monitoring and evaluation mechanisms limits the evidence base attributed to SfD initiatives.

## 5. Conclusion

The paper has highlighted that public health initiatives demand an inter-organisational approach. HIV/AIDS governance as espoused in the UNAIDS “Three Ones” approach showed fragmentation and marginalisation of some sectors, particularly, the sport sector as suggested by the evidence presented. Instead, the multisectoral approach must bring different sectors together, get them involved in the complexities of inter-organisational integration and provide coordination. However, the lack of participation of sport agencies, in particular NSAs at national level has negative implications for the respective NSAs' subnational structures to emulate. Consequently, sport agencies fail to contribute to both national and subnational evidence gathering structures. This strengthens the criticism already made regarding the lack of sport's evidence base to back up the legitimisation of sport as a tool for development. Further weaknesses of sport influencing evidence gathering were revealed in how varied sport as a sector engaged in policy networks, policy communities or issue networks. These variations were influenced by each NSA's political power, internal capabilities or power of resources.

The lack of engagement of NSAs in strategic decision-making tends to exacerbate the lack of influence that sport organisations and the sport sector in general may have in shaping public health policies. The challenge remains for sport policy brokers or programme designers to position themselves within development policy forums to effectively negotiate what the sector can offer to enable health-related behavioural change.

While the influence of SfD NGOs on NSAs in relation to HIV/AIDS programme implementation is widely acknowledged, researching the impact of SfD NGOs on NSAs remains. Further research could look at how partnerships between NSAs and SfD NGOs coordinate evidence gathering to strengthen the legitimisation of sport in development.
